# Tyrosol and Its Analogues Inhibit Alpha-Melanocyte-Stimulating Hormone Induced Melanogenesis

**DOI:** 10.3390/ijms141223420

**Published:** 2013-11-28

**Authors:** Kuo-Ching Wen, Chih-Shiang Chang, Yin-Chih Chien, Hsiao-Wen Wang, Wan-Chen Wu, Chin-Sheng Wu, Hsiu-Mei Chiang

**Affiliations:** 1 Department of Cosmeceutics, China Medical University, Taichung 404, Taiwan; E-Mails: kcwen0520@mail.cmu.edu.tw (K.-C.W.); stacy592@gmail.com (Y.-C.C.); hsesalice@hotmail.com (H.-W.W.); wanjn1024@hotmail.com (W.-C.W.); cswu@mail.cmu.edu.tw (C.-S.W.); 2 Graduate Institute of Pharmaceutical Chemistry, China Medical University, Taichung 404, Taiwan; E-Mail: chihshiang@mail.cmu.edu.tw

**Keywords:** melanogenesis, melanocortin 1 receptor, tyrosol, tyrosol analogues, tyrosinase-related protein

## Abstract

Melanin is responsible for skin color and plays a major role in defending against harmful external factors such as ultraviolet (UV) irradiation. Tyrosinase is responsible for the critical steps of melanogenesis, including the rate-limiting step of tyrosine hydroxylation. The mechanisms of action of skin hypopigmenting agents are thought to be based on the ability of a given agent to inhibit the activity of tyrosinase and, hence, down regulate melanin synthesis. Tyrosol and its glycoside, salidroside, are active components of *Rhodiola rosea*, and in our preliminary study we found that *Rhodiola rosea* extract inhibited melanogenesis. In this study, we examined the effects of tyrosol and its analogues on melanin synthesis. We found that treatment of B16F0 cells to tyrosol (**1**), 4-hydroxyphenylacetic acid (**5**), 3-hydroxyphenylacetic acid (**6**), 2-hydroxyphenylacetic acid (**7**), or salidroside (**11**) resulted in a reduction in melanin content and inhibition of tyrosinase activity as well as its expression. Tyrosol (**1**), 4-hydroxyphenylacetic acid (**5**) and 2-hydroxyphenylacetic acid (**7**) suppressed MC1R expression. Tyrosol (**1**), 4-hydroxyphenylacetic acid (**5**), 3-hydroxyphenylacetic acid (**6**), and 2-hydroxyphenylacetic acid (**7**) inhibited α-MSH induced TRP-1 expression, but salidroside (**11**) did not. All the compounds did not affect MITF and TRP-2 expression. Furthermore, we found that the cell viability of tyrosol (**1**), 4-hydroxyphenylacetic acid (**5**), 3-hydroxyphenylacetic acid (**6**), and 2-hydroxyphenylacetic acid (**7**) at concentrations below 4 mM and salidroside (**11**) at concentrations below 0.5 mM were higher than 90%. The compounds exhibited metal-coordinating interactions with copper ion in molecular docking with tyrosinase. Our results suggest that tyrosol, 4-hydroxyphenylacetic acid, 3-hydroxyphenylacetic acid, 2-hydroxyphenylacetic acid, and salidroside are potential hypopigmenting agents.

## 1. Introduction

Although melanin is essential for protecting skin against UV irradiation damage, abnormal melanin production can lead to hyperpigmentation disorders such as freckles, melanoma, and other types of skin cancer [1–3]. Tyrosinase is a key enzyme in the biosynthesis of melanin and is involved in determining the color of mammalian skin and hair [4,5]. Melanogenesis is regulated by melanin-related enzymes including tyrosinase, tyrosinase-related protein 1 (TRP-1), and tyrosinase-related protein 2 (TRP-2) [4,6]. Ultraviolet (UV) irradiation stimulates the secretion of α-melanocyte-stimulating hormone (α-MSH), which binds to the melanocortin 1 receptor (MC1R). It results in the activation of microphthalmia-associated transcription factor (MITF), which induces the expression of tyrosinase, TRP-1, and TRP-2 [7]. The mechanisms of action of many skin-lightening agents are believed to involve in the down-regulation of melanin synthesis, the inhibition of tyrosinase activity, and inhibition of melanosome transfer [1,5].

**Figure 1.**
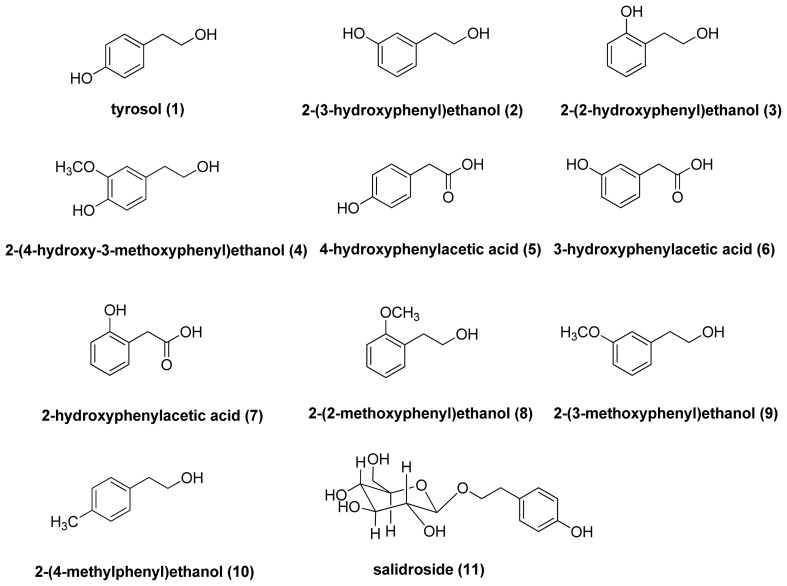
The chemical structures of tyrosol derivatives.

Studies have shown that application of products containing tyrosinase or melanin-inhibiting agents can result in whitening of skin [8,9]. Although several whitening agents such as hydroquinone, arbutin, azelaic acid, retinoic acid, and kojic acid are effective treatments for hyperpigmentation disorders, some of the agents have been shown to have moderate side effects such as dermatitis, skin irritation and melanocyte destruction [10–12]. Thus, there is growing interest in developing hypopigmenting agents from natural sources. Tyrosol (**1**) and its glycoside, salidroside (**11**) (Figure 1), are the major active components of *Rhodiola rosea*, a flowering herb in the Crassulaceae family that has been shown to have anti-aging, anticancer, anti-inflammatory, hepatoprotective, and anti-oxidative effects [13,14]. Tyrosol is readily and dose-dependently absorbed after an oral dose and appears to produce a significant antioxidant effect as well as modest 5-lipoxygenase inhibitory activity *in vivo* [15]. In a previous study, salidroside was shown to inhibit melanin production and tyrosinase activity [16], and the results from our preliminary study indicated that tyrosol inhibited melanin activity. Therefore, compounds similar to tyrosol such as hydroxylphenyl acetic acid may exhibit similar activities on melanogenesis. In addition, hydroxylphenyl acetic acid, which is prevalent in a number of natural products, such as olive oil, bamboo shoots, leaves of Astilbe and roots of dandelion and *Gastrodia elata* have been shown to have anti-inflammatory and antioxidant effects [17–20].

In this study, we investigated the effects and the mechanisms governing the effects of tyrosol and its analogues (Figure 1), namely 2-(3-hydroxyphenyl)ethanol (**2**), 2-(2-hydroxyphenyl)ethanol (**3**), 2-(4-hydroxy-3-methoxyphenyl)ethanol (**4**), 4-hydroxyphenylacetic acid (**5**), 3-hydroxyphenylacetic acid (**6**), 2-hydroxyphenyl acetic acid (**7**), 2-(2-methoxyphenyl)ethanol (**8**), 2-(3-methoxyphenyl)ethanol (**9**), 2-(4-methylphenyl)ethanol (**10**), and salidroside (**11**) on inhibition of melanogenesis in B16F0 mouse melanoma cells.

## 2. Results and Discussion

### 2.1. Results

#### 2.1.1. The Effect of Tyrosol and Its Analogues on Inhibition of Mushroom Tyrosinase Activity

**Figure 2.**
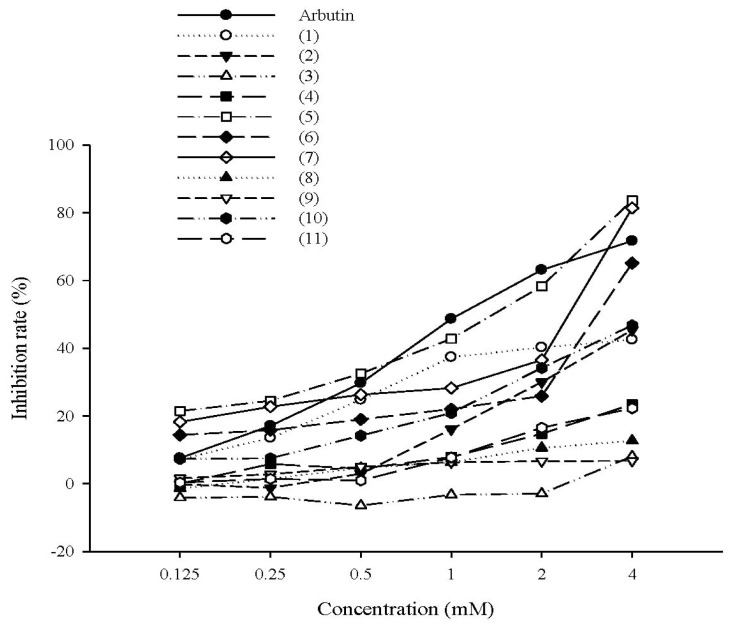
The rate (%) of inhibition of mushroom tyrosinase by tyrosol and its analogues (Tyrosol (**1**); 2-(3-hydroxyphenyl)ethanol (**2**); 2-(2-hydroxyphenyl)ethanol (**3**); 2**-**(4-hydroxy-3-methoxyphenyl)ethanol (**4**); 4-hydroxyphenylacetic acid (**5**); 3-hydroxyphenylacetic acid (**6**); 2-hydroxyphenylacetic acid (**7**); 2-methoxyphenethyl ethanol (**8**); 3-methoxyphenyl ethanol (**9**); 4-methylphenyl ethanol (**10**); and salidroside (**11**). Data are reported as means ± S.D (*n* = 3).

The rates of mushroom tyrosinase inhibition by tyrosol, its analogues, salidroside, and arbutin (positive control) are shown in Figure 2. The rates of tyrosinase inhibition were 42.5% ± 0.7% for tyrosol (**1**), 45.3% ± 1.3% for 2-(3-hydroxyphenyl)ethanol (**2**), 8.1% ± 3.8% for 2-(2-hydroxyphenethyl)ethanol (**3**), 23.4% ± 1.2% for 2-(4-hydroxy-3-methoxyphenyl)ethanol (**4**), 12.7% ± 0.7% for 2-(2-methoxyphenyl)ethanol (**8**), 6.7% ± 0.2% for 2-(3-methoxyphenyl)ethanol (**9**), 46.7% ± 2.7% for 2-(4-methylphenyl)ethanol (**10**) at a concentration of 4 mM, and 22.2% ± 2.1% for salidroside (**11**) at a concentration of 0.4 mM. The IC_50_ values were 1.48 mM for 4-hydroxyphenylacetic acid (**5**), 3.23 mM for 3-hydroxyphenylacetic acid (**6**), 2.60 mM for 2-hydroxyphenylacetic acid (**7**), and 1.30 mM for arbutin. The analogues with higher inhibition rates were 4-hydroxyphenylacetic acid (**5**) (83.6% ± 0.4%), 3-hydroxyphenylacetic acid (**6**) (65.1% ± 1.4%), and 2-hydroxyphenylacetic acid (**7**) (81.3% ± 0.4%), and all exhibited a dose-response effect (Figure 2). According to the results mentioned above, tyrosol (**1**), 4-hydroxyphenylacetic acid (**5**), 3-hydroxyphenylacetic acid (**6**), 2-hydroxyphenylacetic acid (**7**) and salidroside (**11**) were used in the following studies.

#### 2.1.2. The Effect of Tyrosol and Its Analogues on the Viability of B16F0 Cells

**Figure 3.**
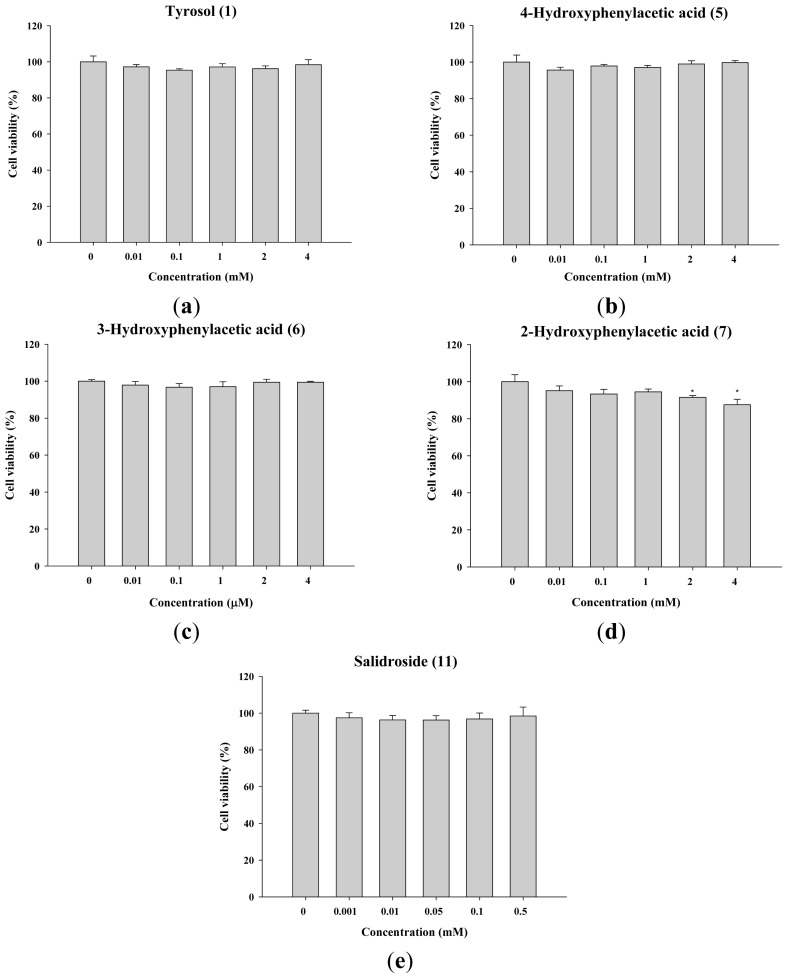
Cell viability (%) of B16 cells exposed to (**a**) tyrosol (**1**); (**b**) 4-hydroxyphenylacetic acid (4-HA) (**5**); (**c**) 3-hydroxyphenylacetic acid (3-HA) (**6**); (**d**) 2-hydroxyphenylacetic acid (2-HA) (**7**) and (**e**) salidroside (**11**). (*p < 0.05 *vs*. Control. Data represent means ± S.D. (*n* = 3)).

The assessment of the cytotoxicity of the compounds is important for developing the compounds as cosmetics. The results of the cell viability assay are shown in Figure 3, and the cell viabilities are higher than 90%. In literature, 80% of cell viability is the criterion for cytotoxicity [21]. Neither tyrosol (<4 mM) nor its analogues exhibited a cytotoxic effect. These results indicated that the cell viabilities of these compounds were acceptable.

#### 2.1.3. Inhibitory Effect of Tyrosol and Its Analogues on Melanin Synthesis in B16F0 Cells

**Figure 4.**
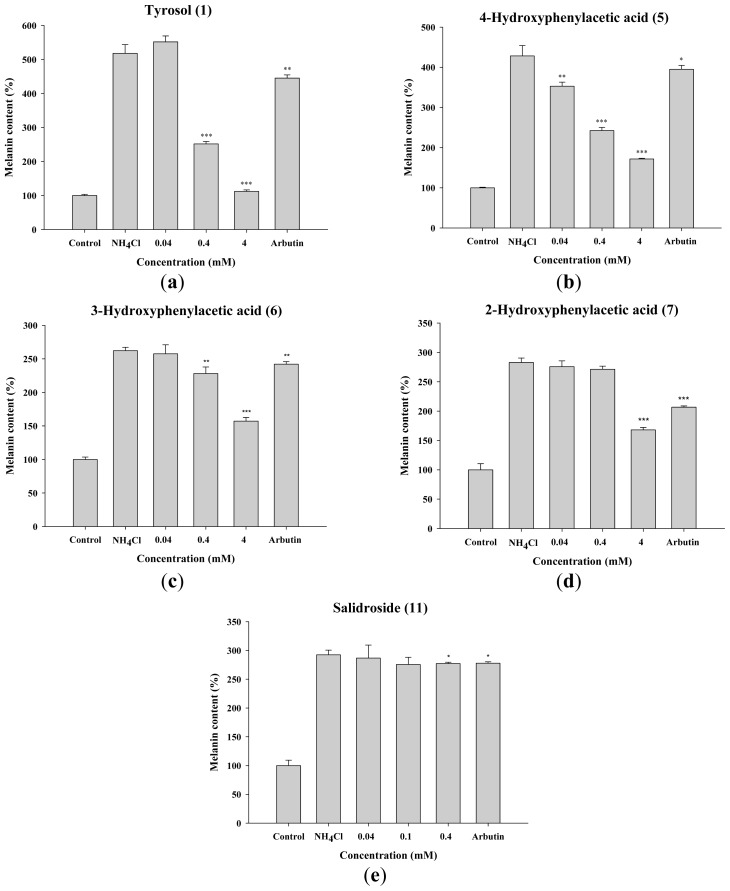
Melanin content (%) in B16F0 cells treated with (**a**) tyrosol (**1**); (**b**) 4-hydroxyphenylacetic acid (4-HA) (**5**); (**c**) 3-hydroxyphenylacetic acid (3-HA) (**6**); (**d**) 2-hydroxyphenylacetic acid (2-HA) (**7**) and (**e**) salidroside (**11**). (*p < 0.05, **p < 0.01, *** *p* < 0.001 *vs*. Control. Data represent means ± S.D. (*n* = 3)).

The effects of tyrosol and its analogues on melanin synthesis in B16F0 cells after stimulation with NH_4_Cl are shown in Figure 4. NH_4_Cl was used as a chemical inducer of melanin synthesis because melanin production and the maturation of melanosomes are regulated by melanosomal pH [22,23]. Tyrosol and its analogues exhibited inhibition of melanin content in a dose-dependent manner. Relative to the control group, melanin content in B16F0 cells was significantly reduced by tyrosol (**1**), 3-hydroxyphenylacetic acid (**6**) and salidroside (**11**) at 0.4 mM, by 4-hydroxyphenylacetic acid (**5**) at 0.04 mM and 2-hydroxyphenylacetic acid (**7**) at 4 mM.

#### 2.1.4. Inhibition of Tyrosinase Activity in B16F0 Cells Exposed to Tyrosol and Its Analogues

**Figure 5.**
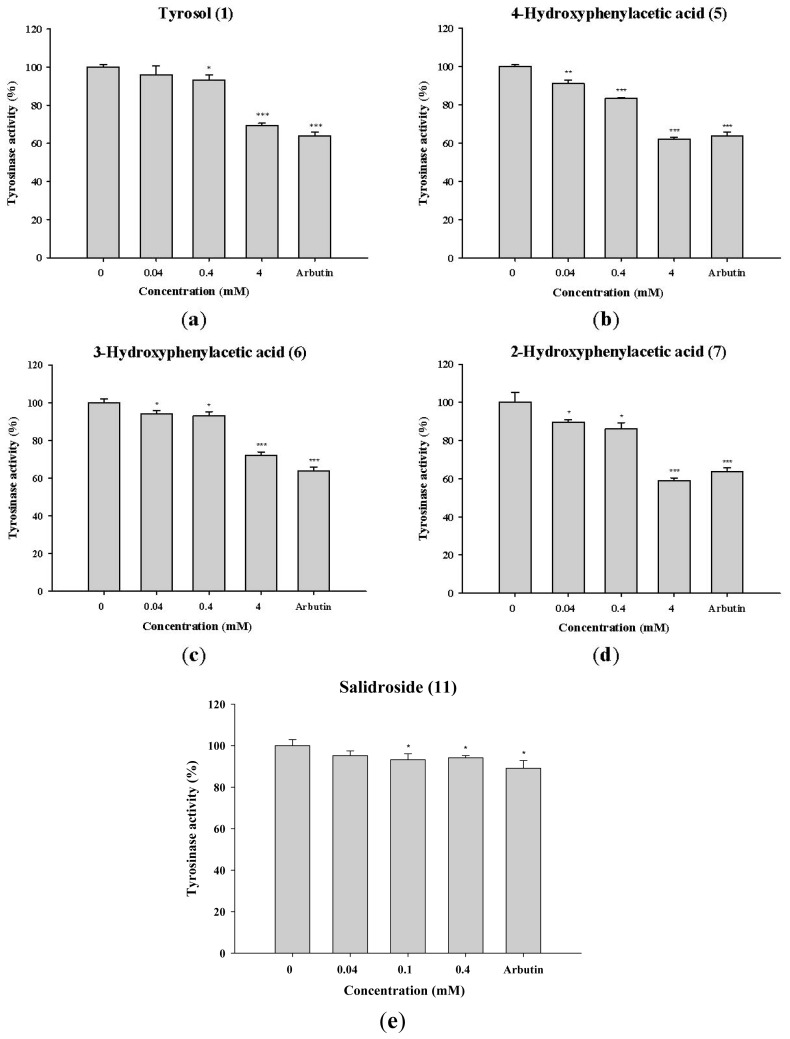
Tyrosinase activity (%) in B16F0 cells exposed to (**a**) tyrosol (**1**); (**b**) 4-hydroxyphenylacetic acid (4-HA) (**5**); (**c**) 3-hydroxyphenylacetic acid (3-HA) (**6**); (**d**) 2-hydroxyphenylacetic acid (2-HA) (**7**) and (**e**) salidroside (**11**). (*p < 0.05, **p < 0.01, ***p < 0.001 *vs*. Control. Data represent means ± S.D. (*n* = 3)).

To determine the effect of tyrosol and its analogues on melanin synthesis, B16F0 cells were treated with various concentrations of tyrosol (**1**), 4-hydroxyphenylacetic acid (**5**), 3-hydroxyphenylacetic acid (**6**), 2-hydroxyphenylacetic acid (**7**), or salidroside (**11**). As shown in Figure 5, all the compounds exhibited significant inhibition of tyrosinase activity in B16F0 cells. The effects of 4-hydroxyphenylacetic acid (**5**), 2-hydroxyphenylacetic acid (**7**) and salidroside (**11**) were similar to the inhibitory effect of arbutin at the same concentration.

#### 2.1.5. Effect of Tyrosol and Its Analogues on MC1R Protein Expression

**Figure 6.**
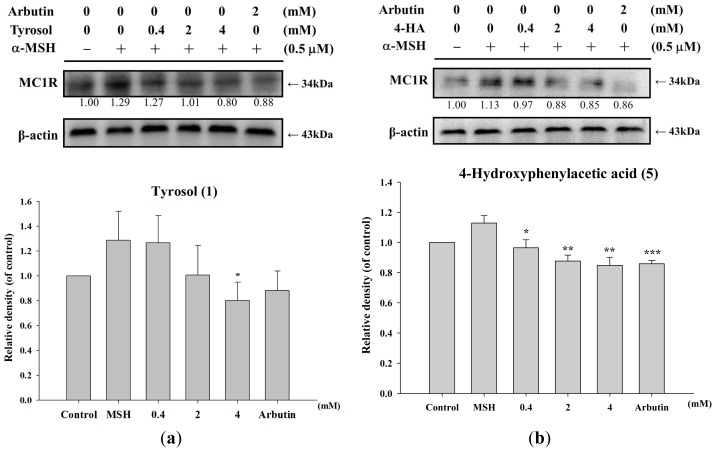

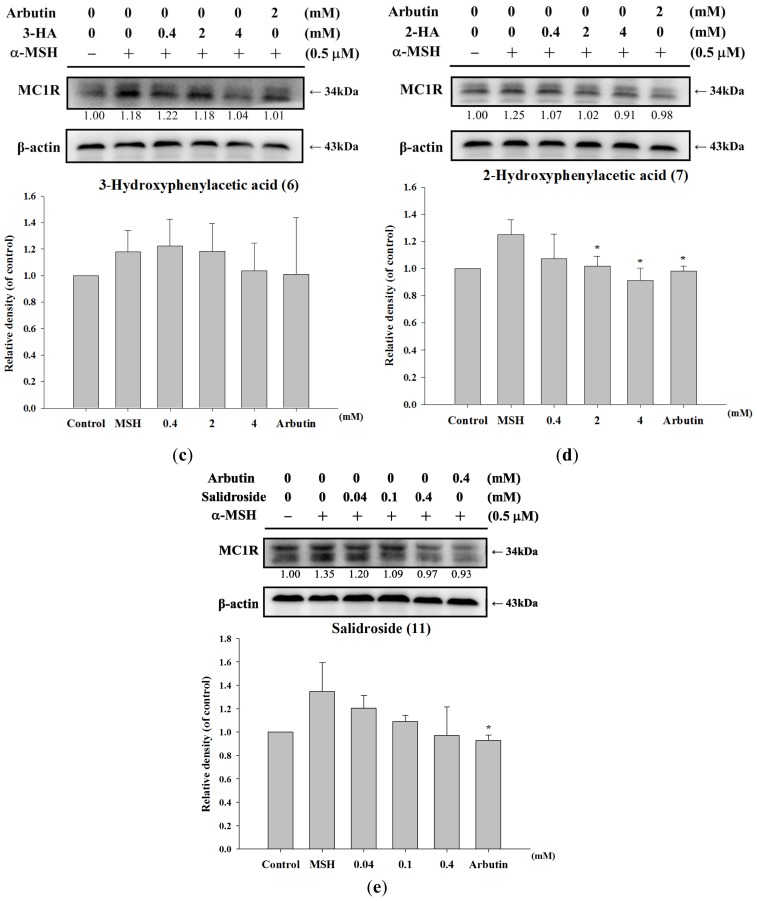
Effect of (**a**) tyrosol (**1**); (**b**) 4-hydroxyphenylacetic acid (4-HA) (**5**); (**c**) 3-hydroxyphenylacetic acid (3-HA) (**6**); (**d**) 2-hydroxyphenylacetic acid (2-HA) (**7**) and (**e**) salidroside (**11**) on protein expression of MC1R in B16F0 cells (*n* = 3). The expression of MC1R protein decreased as the concentration of the treatment increased. (*p < 0.05, **p < 0.01, ***p < 0.001 *vs*. Control.)

MC1R is a transmembrane receptor expressed in melanocytes and melanoma cells. In literature, UV and α-MSH regulates MC1R function at the mRNA and protein levels [24,25]. In this study, tyrosol and its analogues modulated melanogenesis were further examined by measuring the expression of MC1R (34 kDa). As Figure 6 shown, tyrosol (**1**), 4-hydroxyphenylacetic acid (**5**), and 2-hydroxyphenylacetic acid (**7**) suppressed α-MSH-activated MC1R expression in a dose-dependent manner and the activities were similar to the activity of arbutin (Figure 6a,b,d). In addition, although 3-hydroxyphenylacetic acid (**6**) and salidroside (**11**) inhibited α-MSH-activated MC1R expression, there was no significant difference in reduction of MC1R expression between cells in the control group (Figure 6c,e).

#### 2.1.6. Effect of Tyrosol and Its Analogues on Melanogenesis Related Proteins

**Figure 7.**
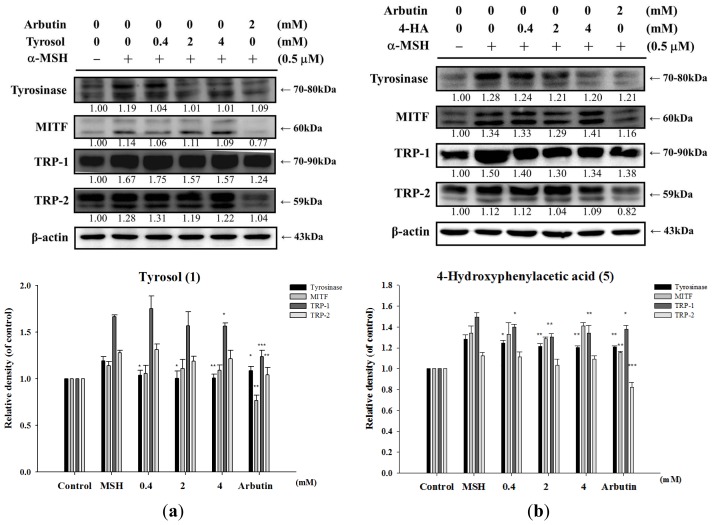

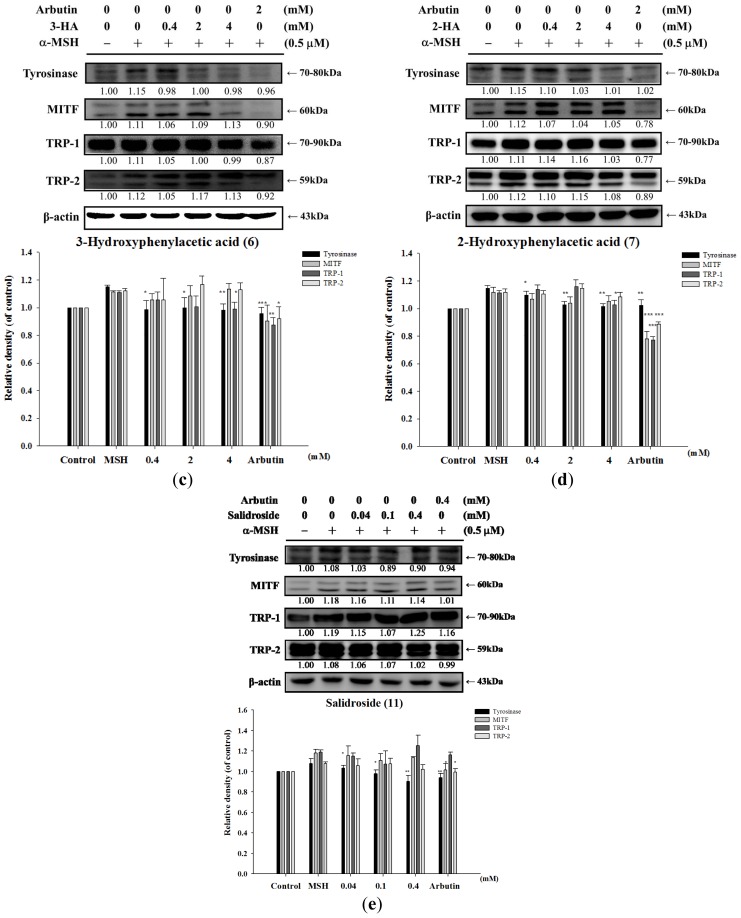
Effects of (**a**) tyrosol (**1**); (**b**) 4-hydroxyphenylacetic acid (4-HA) (**5**); (**c**) 3-hydroxyphenylacetic acid (3-HA) (**6**); (**d**) 2-hydroxyphenylacetic acid (2-HA) (**7**) and (**e**) salidroside (**11**), on α-MSH-induced expression of tyrosinase, MITF, TRP-1, and TRP-2 in B16F0 cells (*p < 0.05, **p < 0.01, ***p < 0.001 *vs*. α-MSH. Data represent means ± S.D. (*n* = 3).

To examine whether the inhibition of melanogenesis by tyrosol and its analogues were related to the expression levels of melanogenesis-related proteins including tyrosinase (70–80 kDa), TRP-1 (70–90 kDa), TRP-2 (59 kDa), and MITF (60 kDa). B16F0 cells were incubated with α-MSH (0.5 μM) and various concentrations of tyrosol (**1**), 4-hydroxyphenylacetic acid (**5**), 3-hydroxyphenylacetic acid (**6**), 2-hydroxyphenylacetic acid (**7**) or salidroside (**11**) for 24 h. Proteins were then separated on 10% SDS-PAGE gels and subjected to western blot analysis. Alpha-MSH would induce tyrosinase, TRP-1, TRP-2 and MITF expression in B16F0 cells (Figure 7). As seen in Figure 7, all the compounds inhibited tyrosinase expression, and four compounds inhibited TRP-1 expression except salidroside. In addition, all the five compounds did not affect MITF and TRP-2 expression (Figure 7a–e). The protein levels of tyrosinase and TRP-1, but not MITF and TRP-2, in α-MSH-stimulated cells were markedly lower in cells that were treated with 4-hydroxyphenylacetic acid (**5**) (2 mM and 4 mM, respectively), than in cells that received other treatments. The effects of 3-hydroxyphenylacetic acid (**6**) and 2-hydroxyphenylacetic acid (**7**) (Figure 7c,d) on the expression of melanogenesis-related proteins were consistent with those elicited by 4-hydroxyphenylacetic acid (**5**) (Figure 7b).

#### 2.1.7. Inhibitory Effect of Tyrosol and Its Analogues on α-MSH Induced Melanin Synthesis in B16F0 Cells

**Figure 8.**
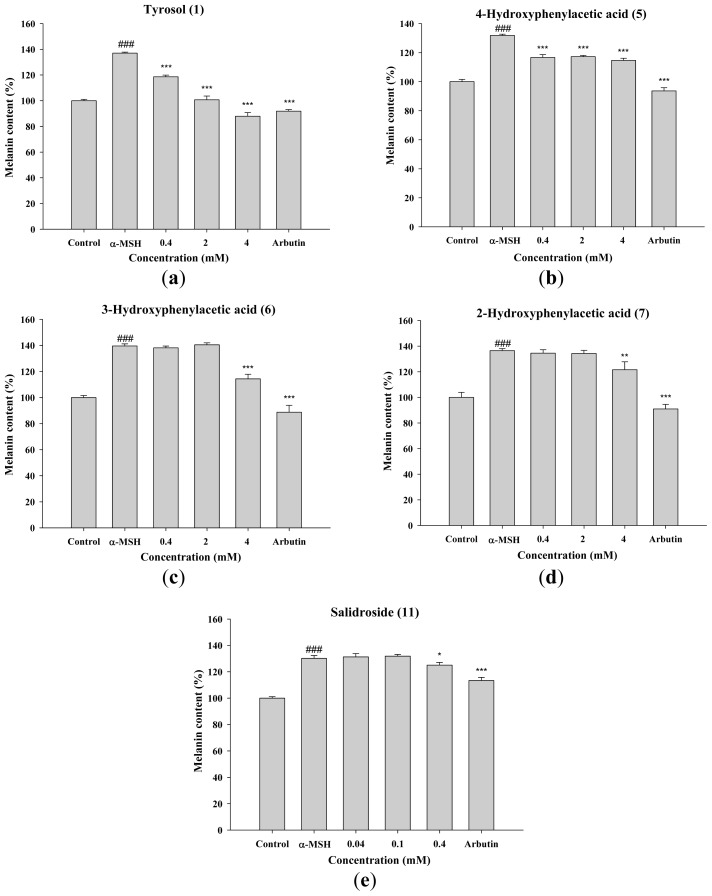
Effects of (**a**) tyrosol (**1**); (**b**) 4-hydroxyphenylacetic acid (4-HA) (**5**); (**c**) 3-hydroxyphenylacetic acid (3-HA) (**6**); (**d**) 2-hydroxyphenylacetic acid (2-HA) (**7**) and (**e**) salidroside (**11**), on α-melanocyte-stimulating hormone (α-MSH) induced melanin content (%) in B16F0 cells. (^###^*p* < 0.001 *vs*. Control. *p < 0.05, **p < 0.01, ***p < 0.001 *vs*. α-MSH. Data represent means ± S.D. (*n* = 3)).

To further confirm the activities of tyrosol and its analogues on melanogenesis, the effects of the compounds on α-MSH induced melanin content in B16F0 were studied. As Figure 8 shown, melanin contents were significantly induced by α-MSH, and tyrosol and its analogues exhibited inhibition of melanin content. Tyrosol (**1**) significantly inhibited α-MSH induced melanin content in B16F0 in a dose-dependent manner (Figure 8a). Tyrosol (**1**), 4-hydroxyphenylacetic acid (**5**), and salidroside (**11**) significantly inhibited α-MSH induced melanin content in B16F0 at 0.4 mM, and 3-hydroxyphenylacetic acid (**6**) and 2-hydroxyphenylacetic acid (**7**) at 4 mM.

#### 2.1.8. Molecular Docking Study

**Figure 9.**
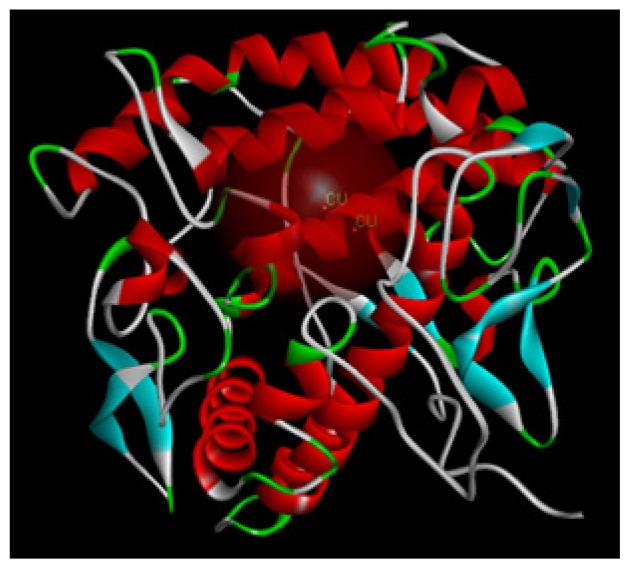
The structure of the H subunit contains a binuclear copper-binding active site in *Agaricus bisporus* mushroom tyrosinase (PDB code 2Y9X).

**Figure 10.**
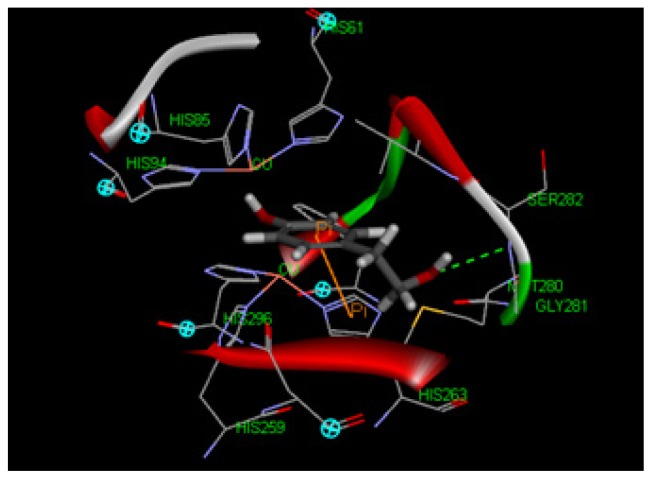
Docking of tyrosol (**1**) into the active site of mushroom tyrosinase. The OH moiety of tyrosol introduced one hydrogen bond with SER282 (green dot line) and its aromatic ring interacted with HIS263 by π–π interaction (orange line). Three histidine residues coordinated each copper ion.

**Figure 11.**
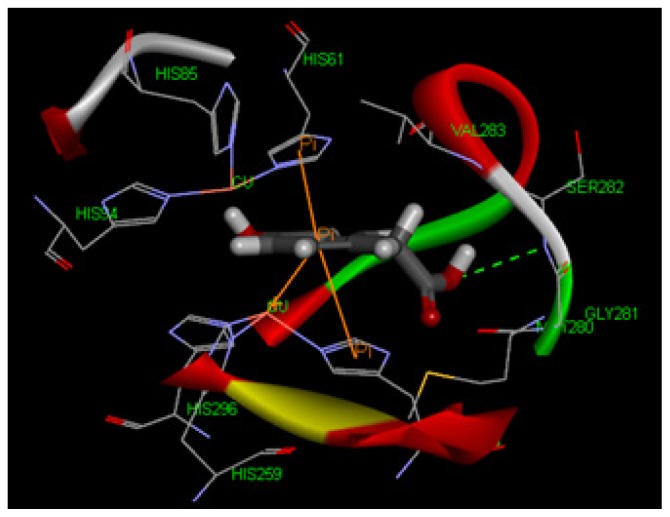
The docking of 4-hydroxyphenylacetic acid (**5**) into the active site of mushroom tyrosinase. The acetic acid moiety of 4-hydroxyphenylacetic acid (**5**) introduced one hydrogen bond with SER282 and its aromatic ring built the π–π interaction with HIS263, HIS61, and copper ion.

**Figure 12.**
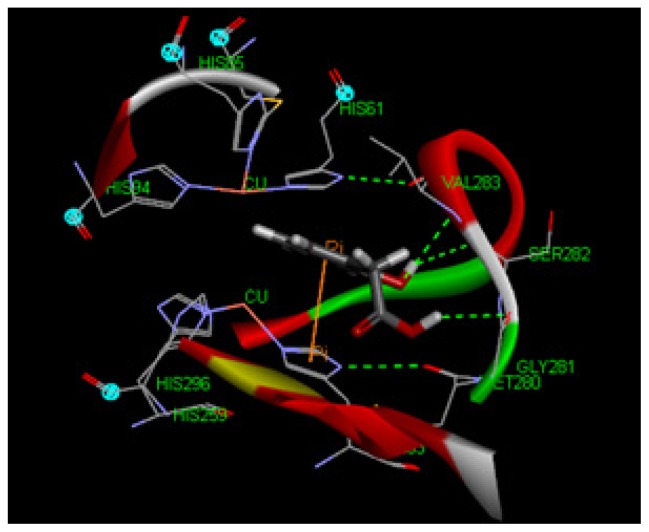
The docking of 2-hydroxyphenylacetic acid (**7**) into the active site of mushroom tyrosinase. The acetic acid moiety of 2-hydroxyphenylacetic acid (**7**) introduced one hydrogen bond with GLY281 and its OH group built two hydrogen-bonds with VAL283 and SER282. The aromatic ring of 2-hydroxyphenylacetic acid (**7**) interacted with HIS263 through π–π interaction.

To explore potential binding of tyrosol and phenylacetic acid derivatives to mushroom tyrosinase, we carried out molecular docking studies focusing on the active site containing binuclear copper ion of H subunit (Figure 9). As the three-dimensional structure of human tyrosinase was unavailable, the *Agaricus bisporus* mushroom tyrosinase was retrieved from the database for the docking study. Tyrosol (**1**) can form a hydrogen bond with SER282 and π–π interaction with HIS263 in the tyrosinase active site (Figure 10). Moreover, 4-hydroxyphenylacetic acid (**5**) forms one hydrogen bond with SER282 and three π–π interactions with HIS263, HIS61, and copper ion (Figure 11). The results indicated that 4-hydroxyphenylacetic acid (**5**) showed a stronger interaction with the active site and possessed potent inhibitory activity. Furthermore, 2-hydroxyphenylacetic acid (**7**) interacted to the active site of mushroom tyrosinase by forming three hydrogen-bond at GLY281, VAL283 and SER282, and π–π interacted to HIS263 (Figure 12). The result shown 2-hydroxyphenylacetic acid (**7**) also possessed good inhibitory activity on tyrosinase compared with 4-hydroxyphenylacetic acid (**5**).

### 2.2. Discussion

Melanogenesis is modulated by a series of enzymes including tyrosinase, TRP-1, TRP-2, and MITF [7,26,27]. Tyrosinase is the rate-limiting enzyme in the process of melanin synthesis and tyrosinase inhibition is the most common approach to achieve skin hypopigmentation [1,4,5,17,26,28,29]. In this study, we found that tyrosol and its analogues inhibited melanin content in B16F0 cells by inhibiting tyrosinase and TRP-1 expression but not TRP-2 or MITF.

The results of this study indicated that tyrosol (**1**), 4-hydroxyphenylacetic acid (**5**), 3-hydroxyphenylacetic acid (**6**), and 2-hydroxyphenylacetic acid (**7**) significantly inhibited melanin synthesis and that the effect was superior to that of arbutin (Figure 4). Because tyrosinase plays a key role in melanogenesis, the effects of tyrosol and its analogues on the activity and expression of this enzyme were measured. The results of this study indicated that tyrosol and its analogues treatment resulted in reduction of tyrosinase activity and expression leading to the down-regulation of melanogenesis (Figures 5 and 7). The reduction in tyrosinase activity by tyrosol and its analogues might be caused either by a direct inhibition of enzyme activity or by a reduction of tyrosinase protein expression in the cells.

It had been reported that tyrosinase activity and melanin synthesis were suppressed at lower pH value [22]. Studies have shown that the suppression of melanin synthesis by some phenolic acids such as ellagic acid [29], tranexamic acid [30], cinnamic acid [31], *p*-coumaric acid [32], and gallic acid [33] and may be due to their ability to acidify melanosomes, thereby inhibiting melanogenesis. In addition, Ito *et al*., reported that the late stages of eumelanogenesis were suppressed by acidic pH and resulted in decrease in tyrosinase activity [34]. Therefore, we speculated that these hydroxylphenyl acetic acid compounds may exhibit melanosomes acidification resulting in melanogenesis inhibition.

The anti-inflammatory and antioxidant effects of hydroxylphenyl acetic acid were reported [19,20]. Oxidative stress and inflammatory factors alter the redox state of cell membrane proteins and disturb melanocyte homeostasis, resulting in melanogenesis [3,6]. Thus, we proposed that the antioxidant and anti-inflammatory activities of tyrosol and its analogues might contribute to their inhibition of melanogenesis. In addition, our finding that 4-hydroxyphenylacetic acid (**5**) was more effective at inhibiting tyrosinase than other compounds might be explained by the fact that the hydroxyl group in that phenol is located at the para-position. Studies have shown that the antioxidant effect of phenolic compounds varies with the position of the substitution on the aromatic ring. For example, compounds with substitution at the para-position, such as 2,4-butylresorcinol (rucinol), have been found to be more effective hydrogen donors and, therefore, better free radical scavengers than compounds with substitution at the meta-position [28,35].

Melanogenesis is regulated by the mitogen-activated protein (MAP) kinase pathway [36]. Upregulation of MAP kinase activates MITF, which then induces the expression of tyrosinase, TRP-1, and TRP-2 [33,37]. In addition to stimulating melanin synthesis, MITF also modulates the differentiation and proliferation of melanocytes [7]. Tyrosinase and TRP-1 were suggested to exhibit 5,6-dihydroxyindole-2-carboxylic acid oxidase promoting melanogenesis [38,39]. TRP-1 has been reported to play an important role in the stabilization of melanosomes. In this study, the inhibition of tyrosol on melanin production was superior to that of arbutin at an equal concentration (4 mM), and that the effect of salidroside was similar to that of arbutin at 0.4 mM. In addition, salidroside and tyrosol significantly inhibited tyrosinase activity, and that the effect of salidroside was similar to that of arbutin at the same concentration. Tyrosol was a more potent inhibitor of cellular tyrosinase activity than salidroside.

Our results indicated that tyrosol suppressed the expression of MC1R, tyrosinase and TRP-1, but had no effect on the expression levels of MITF or TRP-2. Salidroside (0.04 mM), on the other hand, suppressed the expression of tyrosinase, but had no effect on the expression of MC1R, MITF, TRP-1, or TRP-2. 4-Hydroxyphenylacetic acid, 3-hydroxyphenylacetic acid, and 2-hydroxyphenylacetic acid also exhibited inhibition of melanin by suppressing tyrosinase and TRP-1.

Other possible pathway for the down-regulation of melanogenesis by tyrosol and its analogues in B16F0 cells, such as inhibiting maturation of melanosome, tyrosinase gene expression or tyrosinase protein maturation, may not be ruled out based on the results of the present study. More study for determining the other mechanisms of tyrosol and its analogues on reduction of cellular melanin content in B16 cells and animal models are needed in the future.

## 3. Materials and Methods

### 3.1. Materials and Reagents

l-Tyrosine, arbutin, α-MSH, DMSO (dimethyl sulfoxide), 3-(4,5-dimethylthiazol-2-yl)-2,5-diphenyltetrazolium bromide (MTT), tyrosol (**1**), 2-(3-hydroxyphenyl)ethanol (**2**), 2-(2-hydroxyphenyl)ethanol (**3**), 2-(4-hydroxy-3-methoxyphenyl)ethanol (**4**), 4-hydroxyphenylacetic acid (**5**), 3-hydroxyphenylacetic acid (**6**), 2-hydroxyphenyl acetic acid (**7**), 2-(2-methoxyphenyl)ethanol (**8**), 2-(3-methoxyphenyl)ethanol (**9**), and 2-(4-methylphenyl)ethanol (**10**) were purchased from Sigma Chemical Co. (St. Louis, MO, USA). Antibodies recognizing tyrosinase, TRP-1, and TRP-2 were obtained from Santa Cruz Biotechnology, Inc. (Santa Cruz, CA, USA). Antibody recognizing MC1R was purchased from Millipore Corporation. MITF Ab-1(C5) was purchased from Neomarkers Inc. (Fremont, CA, USA).

### 3.2. Inhibitory Effects of Tyrosol and Its Analogues on Mushroom Tyrosinase

The activity of mushroom tyrosinase was determined spectrophotometrically as described in a previous study [40]. l-tyrosine (100 μL) in phosphate buffer saline (pH 6.8) and 80 μL of the same buffer with or without the test sample were added to a 96-well microplate (Nunc, Roskilde, Denmark), to which 20 μL of mushroom tyrosinase (400 U/mL) was added. After the mixture had incubated at 37 °C for 20 min, the amount of dopachrome produced in the reaction mixture was measured in terms of optical density at a wavelength of 492 nm using a microplate reader (Tecan, Grodig, Austria). The inhibitory effects of the test samples on mushroom tyrosinase activity were reported as % inhibition.

The rate of tyrosinase inhibition was calculated using the following equation:

$$\text{Inhibition}(\%)=\frac{\left(A-B)-(C-D\right)}{\left(A-B\right)}\times 100$$

where *A* refers to absorbance with enzyme but without sample, *B* refers to absorbance without enzyme and sample, *C* refers to absorbance with enzyme and sample, and *D* refers to absorbance without enzyme but with sample.

### 3.3. Cell Cultures

B16F0 melanoma cells were purchased from the Food Industry Research and Development Institute (FIRDI) in Taiwan. The cells were cultured in Dulbecco’s modified Eagle’s medium (DMEM) (GIBCO™ Invitrogen CO., Grand Island, NY, USA) supplemented with 10% fetal bovine serum (FBS), 100 units/mL of penicillin, and 100 units/mL of streptomycin in a humidified atmosphere containing 5% CO_2_ in air at 37 °C, as described previously [32].

### 3.4. Cell Viability Assay

The viability of B16F0 melanoma cells was determined by measuring the reduction of 3-(4,5-dimethyl-2-thiazolyl)-2,5-diphenyl-2*H*-tetrazolium bromide (MTT) to formazan as previous study described [41,42]. Briefly, B16F0 melanoma cells were cultured in 96-well plates at 10^4^ cells/well for 24 h. The cells were treated with various concentrations of tyrosol analogues overnight. Then, MTT solution was added to each well. After that, SDS solution was added to dissolve the formazan crystal produced in the cells. The absorbance of each well was then read at 570 nm using a microplate reader (Tecan, Grodig, Austria).

### 3.5. Assay of Cellular Tyrosinase Activity

Cellular tyrosinase activity in B16F0 cells was assayed using l-DOPA as the substrate as previously described [40]. Briefly, B16F0 melanoma cells were plated at a density of 8 × 10^4^ cells/well in a 24-well plate and incubated for 24 h. A 1-mL aliquot of medium containing various concentrations of tyrosol analogues was added and the cells were allowed to incubate for another 24 h. After removing the medium and washing the cells with PBS, 1% (*v*/*v*) Triton X-100 in 50 mM sodium phosphate buffer (pH 6.9) was added and the mixture was freeze-thawed by incubating at −80 °C for 15 min followed by incubation at room temperature for 10 min. The samples were centrifuged at 12,000 × *g* for 15 min. After that, prewarmed freshly prepared substrate (15 mM l-DOPA in 48 mM pH 7.1 sodium phosphate buffer) was added to the supernantant and the mixture was incubated at a 37 °C for 1 h. The absorbance of each well was then read at 405 nm using a microplate reader (Tecan, Grodig, Austria).

The rate of tyrosinase inhibition was calculated using the following equation:

$$\text{Inhibition}(\%)=\frac{{\text{OD}}_{405\text{sample}}}{{\text{OD}}_{405\text{control}}}\times 100$$

### 3.6. The Effect of Tyrosol Analogues on Cellular Melanin Content

B16F0 melanoma cells were seeded at a density of 2 × 10^5^ cells/well in 6-well culture plates and incubated overnight in a humidified atmosphere containing 5% CO_2_ in air at 37 °C. The cells were then treated with medium containing NH_4_Cl, α-MSH, samples, and PBS for 24 h, respectively. The amount of melanin in cell-free culture media was spectrophotometrically measured at 405 nm.

The inhibition rate of melanin was calculated using the following equation:

$$\text{Inhibition}(\%)=\frac{{\text{OD}}_{405\text{sample}}}{{\text{OD}}_{405\text{control}}}\times 100$$

### 3.7. Western Blot Analysis

The cells were harvested and then homogenized with lysis buffer. The cell lysates were centrifuged at 12,000 × *g* for 10 min at 4 °C, and protein content was determined using Bradford reagent (Bio-Rad, Hercules, Berkeley, CA, USA). Proteins (30 μg) were then separated on SDS-PAGE gels and then blotted to a PVDF membrane (Hybond ECL, Amersham Pharmacia Biotech Inc., Piscataway, NJ, USA). Blots were blocked with non-fat milk in TBS buffer containing 0.05% Tween 20 (TBST) and then incubated with specific antibodies, namely MC1R (1:500), MITF (1:100), actin (1:500), TRP-1 (1:500), tyrosinase (1:500), and TRP-2 (1:5000) overnight at 4 °C. The membranes were washed twice with TBST and then incubated with the corresponding conjugated anti-immunoglobulin G-horseradish peroxidase (Santa Cruz Biotechnology Inc., Sabta Cruz, CA, USA). Immunoreactive proteins were detected with an enhanced chemiluminescence plus kit (Fujifilm, LAS-4000, Tokyo, Japan). Signal strengths were quantified using a densitometric program (multi Gauge V2.2).

### 3.8. Molecular Modeling Study

Tyrosinase is a copper-containing enzyme that is widely distributed throughout nature. The enzyme catalyses the oxidation of l-tyrosine to 3,4-dihydroxyphenylalanine (l-DOPA), and further to DOPA quinone. The crystal structure of *Agaricus bisporus* mushroom tyrosinase is well established [43]. This enzyme complex is an H_2_L_2_ tetramer and the H subunit contains a binuclear copper-binding site in the deoxy-state, in which three histidine residues coordinate each copper ion (CU:HIS61, HIS85, HIS94; CU:HIS259, HIS263, HIS296). The established model of the active site was determined using the X-ray structure of *Agaricus bisporus* mushroom tyrosinase (PDB ID:2Y9X) using Discovery Studio 3.1 software. The structure of the full fungal tyrosinase complex was obtained at a 2.3 Å resolution. The 3D structures of ligands were sketched and optimized with smart energy minimization. A LibDock docking algorithm was employed to find the potential binding mode between the enzyme and the phenolic ligand.

### 3.9. Statistical Analysis

The assays are representative of at least three experiments. Values are expressed as mean ± standard deviation (S.D.). Differences in the effect of various treatments were compared by one way ANOVA followed by the Scheffe’s test. A *p-*value < 0.05 was considered to represent statistical significance.

## 4. Conclusions

In this study, based on the results from melanin content and tyrosinase inhibitory assay, we found that tyrosol, 4-hydroxyphenylacetic acid, 3-hydroxyphenylacetic acid, and 2-hydroxyphenylacetic acid salidroside present potential to be applied in skin-whitening cosmetics or therapeutic purpose. These compounds inhibited the process of pigmentation of skin by down-regulating the expression of tyrosinase and TRP-1, but not the expression of MITF and TRP-2.

## Abbreviation

DMEM: Dulbecco’s modified Eagle’s medium
DMSO: dimethyl sulfoxide
FBS: fetal bovine serum
MC1R: melanocortin 1 receptor
MITF: microphthalmia-associated transcription factor
MTT: 3-(4,5-dimethylthiazol-2-yl)-2,5-diphenyltetrazolium bromide
TRP-1: tyrosinase-related protein 1
TRP-2: tyrosinase-related protein 2
α-MSH: α-melanocyte-stimulating hormone
